# Thymoma-associated anti-LGI1 encephalitis and myasthenia gravis: A unique combination with autoantibodies

**DOI:** 10.1016/j.ensci.2022.100395

**Published:** 2022-03-07

**Authors:** Akane Satake, Takamura Nagasaka, Takafumi Kurita, Hiroaki Murata, Takanori Hata, Hiroyuki Shinmura, Hirochika Matsubara, Kazumasa Shindo, Yoshihisa Takiyama

**Affiliations:** a Department of Neurology, Faculty of Medicine, University of Yamanashi, Japan; b Department of Neurology, Kofu Kyoritsu Hospital, Yamanashi, Japan; c Department of Thoracic Surgery, Faculty of Medicine, University of Yamanashi, Japan

**Keywords:** Thymoma, Anti-LGI1 encephalitis, Myasthenia gravis, Myokymia

## Abstract

We report a 77-year-old woman with a thymoma, anti-LGI1antibody associated encephalitis (LGI1 encephalitis), and MG accompanied by positive anti-acetylcholine receptor antibodies (AchR Ab) and anti-titin antibodies (titin Ab). She was treated with thymomectomy followed by immunosuppressive therapy, which resulted in immediate amelioration of motor weakness and gradual improvement of cognitive impairment over the next two years. LGI1 Ab were positive at two months after thymomectomy, followed by negative conversion demonstrated on 1 year examination. The AchR Ab level had gradually decreased but titin Ab was positive on re-examination after two years, although the cognition and motor impairment symptoms had been alleviated. In patients with suspected autoimmune encephalitis, the detection of several autoantibodies including LGI1 and thymomas provides useful information for making an accurate diagnosis.

## Highlights

• A rare case complicated with a thymoma, anti-LGI1antibody associated encephalitis, and myasthenia gravis.
• Accompanied by positive anti-acetylcholine receptor antibodies and anti-titin antibodies.
• The symptoms of encephalitis had improved with thymomectomy and immunosuppressive therapy.
• These unique combinations of the disease and autoantibodies will help us to diagnose and treat autoimmune encephalitis.

## 1 Introduction

Paraneoplastic diseases are defined as autoimmune disorders associated with neoplasms and disorders attributed to biologically active substances secreted from neuroendocrine tumors, often involving multiple organs and tissues.

As for neurological disorders, multiple overlapping neurological syndromes involving the central nervous system (e.g., limbic encephalitis), neuromuscular junctions (e.g., Lambert-Eaton myasthenic syndrome, myasthenia gravis), and the peripheral nervous system (e.g., autonomic neuropathy) are present. Above all, limbic encephalitis is a serious condition affecting the brain that generally requires early treatment. Meanwhile, thymoma is one of the causative tumors that arises in immunoregulatory organs. An investigation on the comorbidity of thymomas showed the complication of single or plural autoimmune diseases including myasthenia gravis (MG) from the perspectives of symptoms and autoantibodies [1]. Of reported thymoma-associated MG patients, around 15% had additional autoimmune diseases [2]. Autoimmune encephalitis (AE) associated with antibodies against leucine-rich glioma inactivated 1 protein (LGI1) is characterized by rapidly developing cognitive impairment, hyponatremia, and hyperintensity of the medial temporal structures on MRI [3]. We report a rare case complicated with a thymoma, anti-LGI1antibody associated encephalitis (LGI1 encephalitis), and MG accompanied by positive anti-acetylcholine receptor antibodies (AchR Ab) and anti-titin antibodies (titin Ab).

## 2 Case report

**Fig. 1:**
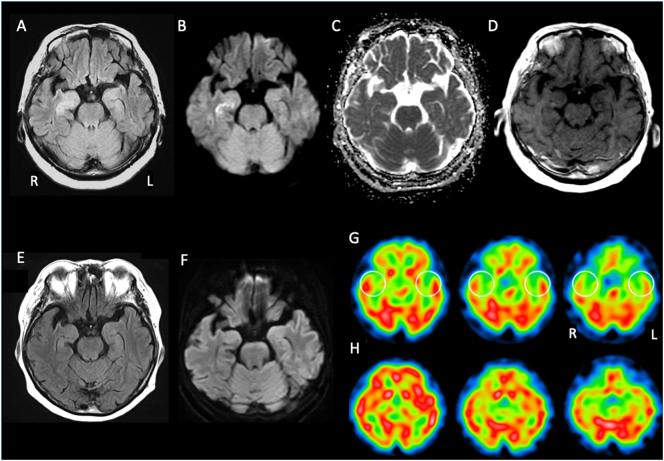
Brain MRI. (A) (B) The right dominant asymmetric hyperintensity involving the bilateral medial temporal regions can be seen in FLAIR and DWI images. (C) The ADC image shows no abnormal intensity corresponding to the hyperintense regions in DWI. (D) A post-contrast T1 image shows no gadolinium enhancement. (E) (F) The high intensity lesions in the medial temporal lobes have disappeared, as seen in FLAIR and DWI images after thymomectomy. Single photon emission computed tomography (SPECT) using I^123^-IMP. (G) Decreased blood perfusion in the bilateral medial temporal lobes can be seen (open circles). (H) Age-matched normal control.

A 77-year-old woman presented with symptoms of memory impairment and involuntary movement of the left face. Two months later, she consulted a hospital with gradually worsening symptoms. Brain MRI revealed left dominant asymmetric hyperintensity involving the bilateral medial temporal regions in FLAIR and DWI sequences (Fig. 1 A, B). The ADC image showed no abnormal intensity corresponding to the hyperintense regions in DWI sequences (Fig. 1 C). A post-contrast T1 image showed no gadolinium enhancement (Fig. 1 D). CSF analysis revealed normal cell counts (lymphocytes, 1/μl; normal range, 5>), and protein (29 mg/dL; normal range, 10–40) and glucose levels (57 mg/dL; normal range, 50–75), and no evidence of infection, the absence of cytomegalovirus, herpes simplex virus and varicella zoster virus specific IgG and IgM being confirmed by EIA. She was diagnosed as having suspected limbic encephalitis and thus treated with methylprednisolone (mPSL) pulse therapy (1 g per day for three days). Thereafter, she was transferred to our hospital for further treatment four months after the onset of symptoms.

**Fig. 2:**
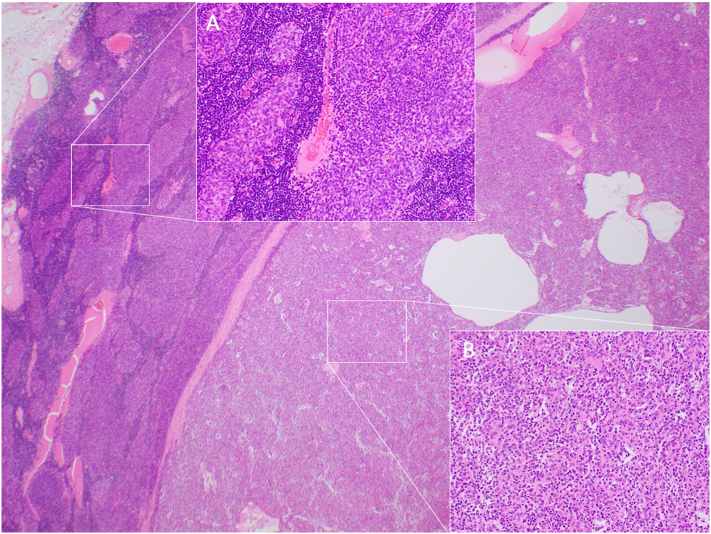
Histological findings for the thymoma, HE staining x20. A combined thymic epithelial tumor, approximately 30% of WHO Type B3 (A) and 70% of Type A (B), can be seen.

On admission, she had mild cognitive impairment and mild bilateral ptosis, and left dominant facial weakness with myokymia (Video 1). The muscular strength of the limbs was normal but there was slight fatigability. Serum sodium was normal (134 mmol/L). Serum antibody tests were positive for LGI1 (indirect immunofluorescence method), AchR (4.8 nmol/L; normal range, 0.2 nmol/L>), and titin (3+, immunoblotting), and negative for anti-CASPR2, amphiphysin, CV2, Ma2, and Yo antibodies (Ab). We did not perform autoantibody studies on CSF. Chest contrast-enhanced CT revealed a thymoma. Single photon emission computed tomography (SPECT) using I^123^-IMP showed decreased blood perfusion in the bilateral medial temporal lobes (Fig. 1 G). EEG showed a normal basic rhythm without epileptic discharges. The bilateral ptosis improved after injection of tensilon, but repetitive stimulation testing of the orbicularis oculi and biceps brachii was normal.

She was diagnosed as having a thymoma, LGI1 encephalitis and MG, and was treated with second mPSL pulse therapy followed by oral prednisolone and thymomectomy. We confirmed that the thymoma was a combined thymic epithelial tumor pathologically (Fig. 2). One month after the thymomectomy, the facial myokymia and ptosis were alleviated (Video 1), and the high intensity lesions in the medial temporal lobes were resolved, as seen on MRI (Fig. 1 E; FLAIR, F; DWI). Also, her cognitive dysfunction including recent memory loss continued to improve gradually over the next two years after thymomectomy, the treatment with oral prednisolone being tapered down to 10 mg/day slowly. Concerning changes in serum antibodies, LGI1 Ab were positive at two months after thymomectomy, followed by negative conversion demonstrated on 1 year examination. The AchR Ab level had gradually decreased to 0.2 nmol/L, but titin Ab were positive (cytometric cell-based assays) on re-examination after 2 years, although the cognition and motor impairment symptoms had been alleviated.

Authors have received and obtained patient consent for video recording and publication.

## 3 Discussion

Paraneoplastic neurological syndromes (PNS) are disorders resulting from a remote effect of a neoplasm. In recent years, PNS became recognized to include non-paraneoplastic conditions, that is, disorders attributed to the production of autoantibodies without neoplasms. The pathogenic basis in our patient is the PNS in association with a thymoma revealed by the detection of autoantibodies. A report showed that 55% of 85 thymoma patients had at least 1 paraneoplastic manifestation, with more than 2 in 7%, and 39% had at least MG. Whereas, LGI1 Ab positive disorders are few and their association with thymomas is rarely observed, i.e., in only 2.8% [4]. Another report showed that among 43 patients with thymoma-associated AE, 3% were positive for anti-LGI1 Ab [5]. Thus, the combination of LGI1 encephalitis, MG and a thymoma is very infrequent, only one case having been reported previously [6].

In our patient, titin Ab were found other than LGI1 Ab and Ach R Ab. Titin Ab reflect the paraneoplastic property associated with thymomas in MG for the most part, and are also seen in a small portion of patients with paraneoplastic diseases other than MG [7]. Only three cases of thymoma-associated AE positive for titin Ab have been reported [[8], [9], [10]]. In our case, the symptoms due to encephalitis were alleviated with negative conversion of LGI1 Ab on immunosuppressive therapy, although titin Ab continued to be positive. Therefore, it is thought that titin Ab are unrelated to encephalitis.

As for the treatment in our case, the first mPSL pulse therapy performed in a previous hospital provided partial improvement, which was moderate for myasthenia and mild for cognitive impairment, but did not stop the progression of the disease. So, we thought that combination therapy of corticosteroids and thymomectomy could control the symptoms. Accordingly, we performed the second mPSL pulse therapy to stabilize her general condition just before thymomectomy, followed by tapering down of oral prednisolone slowly. A recent retrospective study indicated that corticosteroids are more effective than intravenous immunoglobulin (IVIG) for treatment of LGI1 encephalitis in the acute phase [11]. Therefore, LGI1 encephalitis without a thymoma can be treated with corticosteroids. In the current diagnostic criteria for paraneoplastic neurological syndromes, LGI1 Ab are classified as lower risk antibodies related to limbic encephalitis [12]. From this point of view and the mild myasthenic symptoms, we were able to accomplish overall medication without non-steroidal immunosuppressants, IVIG or plasma exchange along with thymomectomy. In conclusion, it is significant that there are sufficient therapeutic effects with the thymomectomy for autoimmune disorders associated with thymomas, which are thought to be the cause of the autoantibody production.

In our patient, faciobrachial dystonic seizures (FBDS), a characteristic feature of LGI1 encephalitis, was not observed, but myokymia, a symptom of other LGI1 Ab positive disorders including Issacs syndrome and Morvan syndrome, was observed in her face. This complication in LGI1 encephalitis has not been previously reported.

In thymoma patients, we should be aware of several complications of autoimmunity. Also, we need to confirm the presence of thymomas and LGI1 Ab in patients with suspected autoimmune encephalitis including limbic encephalitis.

The following is the supplementary data related to this article.

Video 1 Myokymia in her left face and bilateral blepharoptosis can be observed (first half of video, pre-treatment). The symptoms were alleviated after thymomectomy (second half, post-treatment). Video 1

## Declaration of Competing Interest

The authors declare that the research was conducted in the absence of any commercial or financial relationships that could be construed as a potential conflict of interest.
